# Influence of sex-specific concurrent changes in age, maturity status, and morphological covariates on the development of peak ventilatory variables in 10–17-year-olds

**DOI:** 10.1007/s00421-020-04569-1

**Published:** 2020-12-07

**Authors:** Neil Armstrong, Jo Welsman

**Affiliations:** Children’s Health and Exercise Research Centre, University of Exeter, St Lukes Campus, Heavitree Road, Exeter, EX1 2LU UK

**Keywords:** Breathing frequency, Morphological variables, Multiplicative allometric modelling, Paediatric norms, Pulmonary ventilation, Tidal volume

## Abstract

**Purposes:**

(i) To investigate the influence of concurrent changes in age, maturity status, stature, body mass, and skinfold thicknesses on the development of peak ventilatory variables in 10–17-year-olds; and, (ii) to evaluate the interpretation of paediatric norm tables of peak ventilatory variables.

**Methods:**

Multiplicative multilevel modelling which allows both the number of observations per individual and the temporal spacing of the observations to vary was used to analyze the expired ventilation (peak ${\dot{\mathrm{V}}}_{\mathrm{E}}$) and tidal volume (peak *V*_T_) at peak oxygen uptake of 420 (217 boys) 10–17-year-olds. Models were founded on 1053 (550 from boys) determinations of peak ventilatory variables supported by anthropometric measures and maturity status.

**Results:**

In sex-specific, multiplicative allometric models, concurrent changes in body mass and skinfold thicknesses (as a surrogate of FFM) and age were significant (*p* < 0.05) explanatory variables of the development of peak ${\dot{\mathrm{V}}}_{\mathrm{E}}$, once these covariates had been controlled for stature had no additional, significant (*p* > 0.05) effect on peak ${\dot{\mathrm{V}}}_{\mathrm{E}}$. Concurrent changes in age, stature, body mass, and skinfold thicknesses were significant (*p* < 0.05) explanatory variables of the development of peak *V*_T_. Maturity status had no additional, significant (*p* > 0.05) effect on either peak ${\dot{\mathrm{V}}}_{\mathrm{E}}$ or peak *V*_T_ once age and morphological covariates had been controlled for.

**Conclusions:**

Elucidation of the sex-specific development of peak ${\dot{\mathrm{V}}}_{\mathrm{E}}$ requires studies which address concurrent changes in body mass, skinfold thicknesses, and age. Stature is an additional explanatory variable in the development of peak *V*_T_, in both sexes. Paediatric norms based solely on age or stature or body mass are untenable.

## Introduction

Paediatric cardiopulmonary exercise tests (CPETs) are widely used to assess cardiovascular and pulmonary health (Rowland 2018), and incremental CPETs with respiratory gas analyses are internationally recognized as the ‘gold standard’ for evaluation of both healthy youth (American Thoracic Society/American College of Chest Physicians 2003) and those with pulmonary diseases (Lang et al. 2020). Oxygen uptake ($\dot{\mathrm{V}}{\mathrm{O}}_{2}$) at the termination of an incremental CPET to voluntary exhaustion (i.e., peak $\dot{\mathrm{V}}{\mathrm{O}}_{2}$) is the most comprehensively researched variable in the history of paediatric exercise science (Falk et al. 2018). In contrast, although first reported over 80 years ago (Robinson 1938), the documentation of corresponding peak ventilatory variables (i.e., values at peak $\dot{\mathrm{V}}{\mathrm{O}}_{2}$) is relatively sparse, and their development in youth in relation to concurrent changes in age, growth, and maturation has not been effectively addressed.

Tables of age- and body size-related paediatric norms for peak ventilatory variables are readily available and routinely used for comparative analyses of healthy youth and as a benchmark for those with pulmonary diseases (e.g., Bongers et al. 2014). Body mass and stature change concurrently with age, growth, and maturity status but at different rates (Baxter-Jones 2017). Published paediatric norm values of peak ventilatory variables referenced solely against age or body mass or stature, therefore, make a limited contribution to describing the development of peak ventilatory responses to exercise in relation to growth and maturation (Blais et al. 2015).

Collectively, cross-sectional (e.g., Ondrak and McMurray 2006; Prado et al. 2010; Giardini 2011) and longitudinal studies (e.g., Rutenfranz et al. 1981; Kemper and Vershuur 1985; Rowland and Cunningham 1997) have consistently reported peak expired ventilation (peak ${\dot{\mathrm{V}}}_{\mathrm{E}}$) and peak tidal volume (peak *V*_T_) to increase with age in both sexes. Studies have generally attempted to control for growth by dividing peak ${\dot{\mathrm{V}}}_{\mathrm{E}}$ and peak *V*_T_ by body mass (ratio scaling) and analyzing them in L·kg^−1^·min^−1^ and L·kg^−1^, respectively. There are conflicting reports, but, on balance, it appears that there is no change in boys’ age-related ${\dot{\mathrm{V}}}_{\mathrm{E}}$·kg^−1^ and no change or a slight decline in girls’ age-related ${\dot{\mathrm{V}}}_{\mathrm{E}}$·kg^−1^, with *V*_T_·kg^−1^ remaining stable with age in both sexes. Peak breathing frequency (peak *f*_b_) is reported as only marginally influenced by both age and morphological covariates, with no consistent sex differences. However, controlling for growth through ratio scaling assumes an underlying set of specific statistical assumptions which are seldom (if ever) met in paediatric exercise studies (Welsman and Armstrong 2019) and has confused understanding of developmental exercise physiology for over 80 years (Armstrong and Welsman 2020a).

In an innovative cross-sectional study of 76 10–15-year-old boys, Mercier et al. (1991) found that fat-free mass (FFM) estimated from body mass and skinfold thicknesses, explained a greater percentage of the variance in both peak ${\dot{\mathrm{V}}}_{\mathrm{E}}$ and peak *V*_T_ than either body mass or stature. Recognizing the fallacy of ratio scaling, the authors investigated the relationships between peak ${\dot{\mathrm{V}}}_{\mathrm{E}}$ and peak *V*_T_ with body mass, stature, and estimated FFM, using what they referred to as the ‘simple allometric equation’, where *y* = *a*·*x*^b^. Ratio scaling imposes the mathematical relationship *y* = *a*·*x*^1.0^ upon the physiological variable (*y*) and body size (*x*), whereas allometric modelling allows the body size exponent (b) to be derived from the data set under investigation. Mercier et al. (1991) demonstrated that ratio scaling did not effectively control for morphological covariates and concluded that FFM was the appropriate morphological covariate to ‘normalize’ peak ${\dot{\mathrm{V}}}_{\mathrm{E}}$ during growth. However, their ‘simple’ allometric analyses only considered a single morphological covariate at a time.

In a mixed cross-sectional-longitudinal extension of Mercier et al.’s (1991) study, Prioux et al. (1997) split 44 of the boys into three age groups (11.2, 12.9, and 14.9 years at onset) and tested them on three annual occasions. The authors confirmed their earlier observation that estimated FFM was the major morphological determinant of both peak ${\dot{\mathrm{V}}}_{\mathrm{E}}$ and peak *V*_T_. They also noted that the individual relationships of body mass, stature, and particularly FFM with peak ventilatory variables changed during growth and maturation. The data provided, however, few insights into the effects of concurrent changes in age, body mass, FFM, and stature on the development of peak ventilatory variables.

Cross-sectional studies only provide a single ‘snapshot’ in time of a continuous process and longitudinal peak ventilatory data have consistently been reported as a series of ‘snapshot’, age-related, ratio-scaled values. The development of peak ventilatory variables in youth, therefore, remains to be explored using an appropriate longitudinal model. The emergence (Aitkin et al. 1981) and development (Rasbash et al. 2018) of multilevel regression modelling and the application of multiplicative allometric modelling to the developmental exercise physiology of trained (Nevill et al. 1998) and untrained youth (Armstrong et al. 1999) have opened up new avenues of research. Multiplicative allometric modelling enables the effects of age, maturity status, and several morphological covariates to be partitioned concurrently within an allometric framework, to provide a flexible and sensitive interpretation of the development of responses to exercise. No previous study has applied this technique to explain the development of peak ventilatory responses to exercise.

The purposes of the present investigation are therefore, (i) to use multiplicative allometric modelling to enhance understanding of the sex-specific influence of concurrent changes in age, maturity status, body mass and skinfold thicknesses (as a surrogate for FFM), and stature on the development of peak ventilatory variables from 10–17 years of age; and, (ii) to evaluate the use of current paediatric norms to interpret CPET data on the development of peak ventilatory variables in youth.

## Methods

### Participants

Initially, 234 (115 girls and 119 boys) students, aged 10–11 years, from local state schools volunteered to participate in a longitudinal study of aerobic (or cardiopulmonary) fitness involving three annual testing occasions. The project was subsequently extended to cover the range 10–17 years, with an additional 186 (88 girls and 98 boys) students, aged 11–14, volunteering to join the project. This resulted in 1053 determinations of 10–17-year-old’s peak ventilatory variables. The project received ethical approval from the District Health Authority Ethical Committee and all participants provided written informed consent signed by themselves and their guardian. Multiplicative allometric models of aerobic fitness data have been reported elsewhere (Armstrong and Welsman 2020a), but the corresponding ventilatory data have not been previously modelled or reported.

### Experimental procedures

#### Determination of resting variables

Participants were well habituated to the laboratory environment and to the experimental procedures prior to the study. Chronological age was computed from date of birth and date of examination. Anthropometric measures were taken as recommended by the International Biological Programme (Weiner and Lourie 1981) and apparatus was calibrated according to the manufacturer’s instructions. Stature was measured using a Holtain stadiometer (Holtain, Crmych, Dyfed, UK) and body mass was determined using Avery balance scales (Avery, Birmingham, UK). Skinfold thicknesses over the triceps and subscapular regions were measured using Holtain skinfold calipers. Maturity status was visually assessed by the Research Centre nurse using the indices for pubic hair (PH) development described by Tanner (1962).

#### Determination of exercise variables

Following a standardized warm-up, peak $\dot{\mathrm{V}}{\mathrm{O}}_{2}$, peak ${\dot{\mathrm{V}}}_{\mathrm{E}}$, peak *V*_T_, peak *f*_b_, and peak heart rate (peak HR) were determined during a progressive, incremental CPET to voluntary exhaustion on a motorised treadmill (Woodway, Cranlea Medical, Birmingham, UK). Throughout each CPET, HR, ${\dot{\mathrm{V}}}_{\mathrm{E}}$, *V*_T_, *f*_b_, and inspired and expired respiratory gases were monitored continuously using an electrocardiograph and an Oxycon Sigma on-line gas-analysis system (Cranlea Medical,) which was calibrated prior to each test using a range of flow rates with a 3 L Hans Rudolph calibration syringe and gases of verified concentration. Depending on age, the CPET commenced at a treadmill belt speed of either 1.94 m·s^−1^ (7 km·h^−1^) or 2.22 m·s^−1^ (8 km·h^−1^), and increased by 0.28 m·s^−1^ (1 km·h^−1^) for each 2–3-min stage until a speed of 2.78 m·s^−1^ (10 km·h^−1^) was reached. Subsequently, belt speed was held constant and the gradient was incrementally increased by 2.5% until voluntary exhaustion. The highest 30 s $\dot{\mathrm{V}}{\mathrm{O}}_{2}$ attained was recorded as peak $\dot{\mathrm{V}}{\mathrm{O}}_{2}$ and accepted as a maximal index if clear signs of intense exertion (e.g., hyperpnea, facial flushing, unsteady gait, and profuse sweating) were demonstrated, supported by an HR which was levelling-off over the final stages of the test at a value within 5% of the mean maximal HRs we have previously reported for boys and girls of these ages (Armstrong et al. 1991). All participants included herein satisfied these criteria on each test occasion and the corresponding ${\dot{\mathrm{V}}}_{\mathrm{E}}$, *V*_T_, and *f*_b_ were recorded as peak ventilatory values.

## Data analyses

Descriptive data were analysed using SPSS (v24 IBM) and factors associated with the development of peak ventilatory variables were analysed using multiplicative allometric modelling (MLWin v3.02, Centre for Multilevel Modelling, University of Bristol, UK).

Sex-specific peak ${\dot{\mathrm{V}}}_{\mathrm{E}}$, peak *V*_T_, and peak *f*_b_ were graphed against age, body mass, and stature. For illustrative purposes, FFM was estimated using the youth-specific equations reported by Slaughter et al. (1988) and graphed against peak ventilatory variables. In the multiplicative allometric models, the sum of triceps and subscapular skinfolds in conjunction with body mass was used as a surrogate for FFM, rather than rely on predictions from equations likely to be population-specific (Roemmich et al. 1997). In accord with recent multiplicative allometric models of the development of other exercise variables (Armstrong and Welsman 2019a, 2019b,2019c,2020b; Armstrong et al., 2019), the use of body mass and sum of skinfolds as a surrogate of FFM produced multiplicative allometric models with a significantly (*p* < 0.05) better statistical fit than those using youth-specific equations to estimate FFM and it is these models which are reported herein.

In contrast to traditional analysis techniques for repeated measurements, the multilevel regression modelling procedure is not constrained by restrictive data requirements such as discrete measurement occasions and complete data sets for each individual. Both the number of measurement occasions and the temporal spacing of the measurements may vary between individuals. Multilevel modelling describes not only underlying trends in a response (the fixed part of the analysis) but also concurrently models the variation around this mean response at both levels of the analysis (the random structure) (Rasbash et al. 2018). The present study adopted a multiplicative allometric modelling approach which has been demonstrated to be appropriate for body size-related exercise performance data which tend to be skewed with heteroscedastic residuals (Nevill et al. 1998, 2005; Welsman and Armstrong 2000).

The baseline models are described in Eqs. 1 and 2, where y is the physiological variable (i.e., either peak ${\dot{\mathrm{V}}}_{\mathrm{E}}$ or peak *V*_T_):

1 $$ y = {\text{ body mass}}^{{\text{k}}} \cdot \, \exp \, \left( {a_{j} + b \, \cdot{\text{ age}} + c \, \cdot{\text{ age}}^{2} } \right)\varepsilon_{ij} . $$

Log transformation linearized the model to form the starting point for analyses:

2 $$ \log_{e} y = k\cdot\log_{e} {\text{body mass }} + a_{j} + b\cdot{\text{age }} + \, c\cdot{\text{age}}^{{2}} + \, \log_{e} \left( {\varepsilon_{ij} } \right). $$

All investigated parameters were fixed with the exception of the constant (*a*, intercept term) which was allowed to vary randomly at level 2 (between individuals) and the multiplicative error term ε which also varied randomly at level 1 (within individuals). The subscripts *i* and *j* denote this random variation at levels 1 and 2, respectively. Age was centred on the group mean. From the baseline model including age and body mass, additional variables (sum of skinfold thicknesses and stature) were explored. In subsequent analyses, maturity status (i.e., PH stages 2, 3, 4, and 5 compared to PH stage 1) was introduced as an additional indicator variable.

Parameter estimates were considered significant (*p* < 0.05) where their value exceeded 2 × the standard error (S.E) and only significant terms were included in the final models. The change in deviance statistic (− 2 × log-likelihood) was used to assess the goodness of fit of the models. A comparison of the goodness of fit of the different models was obtained from the change in the deviance statistic (− 2 × log-likelihood) with reference to the number of fitted parameters. In a comparison of two models with the same number of fitted parameters, the model with the smallest − 2 × log-likelihood reflects that with the best fit. Additional parameters contribute to improved fit from the change in − 2 × log-likelihood according to a chi squared statistic for additional degrees of freedom added.

## Results

**Fig. 1:**
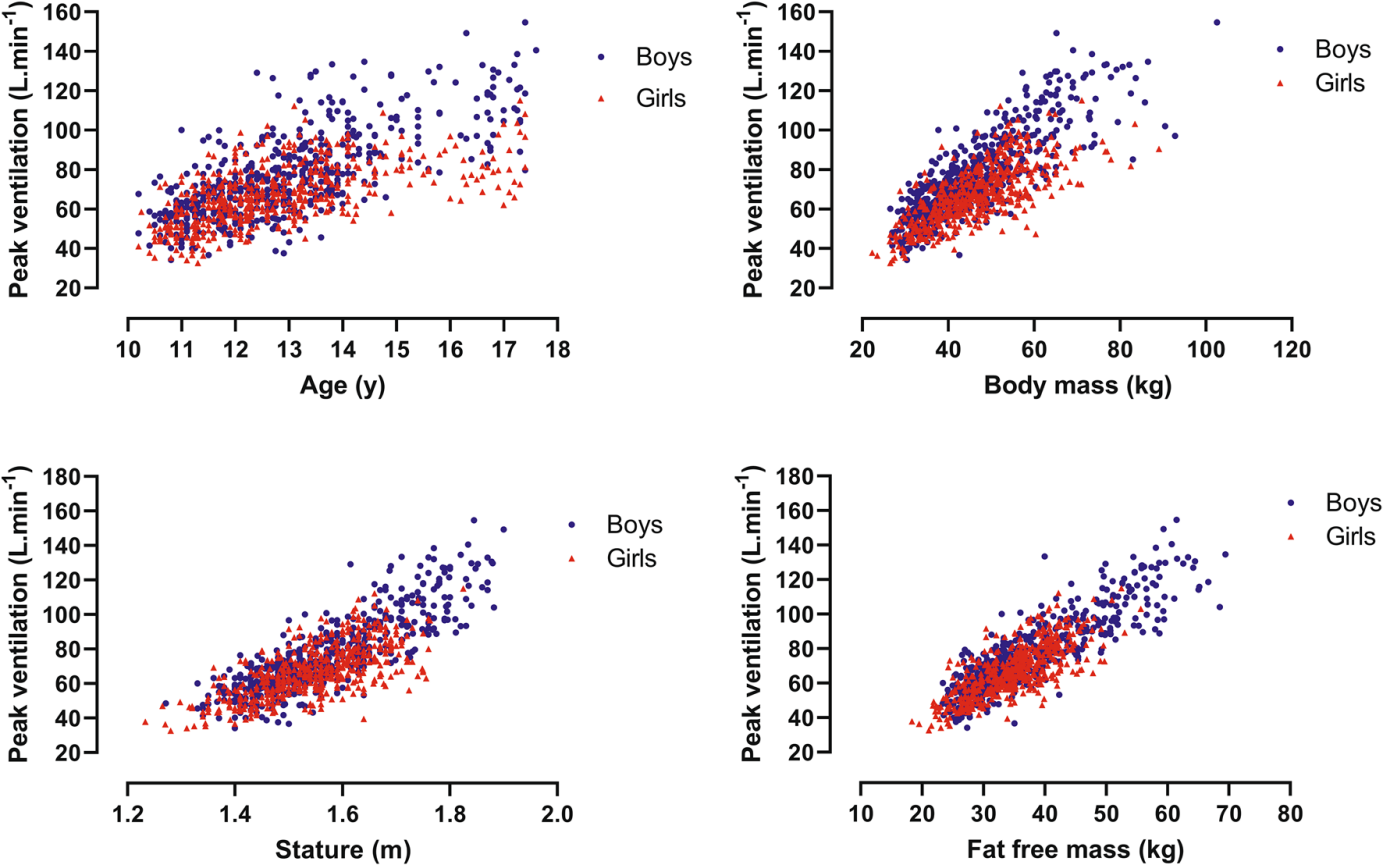
Peak expired ventilation in relation to age, body mass, stature, and estimated fat-free mass by sex in 10–17-year-olds

**Fig. 2:**
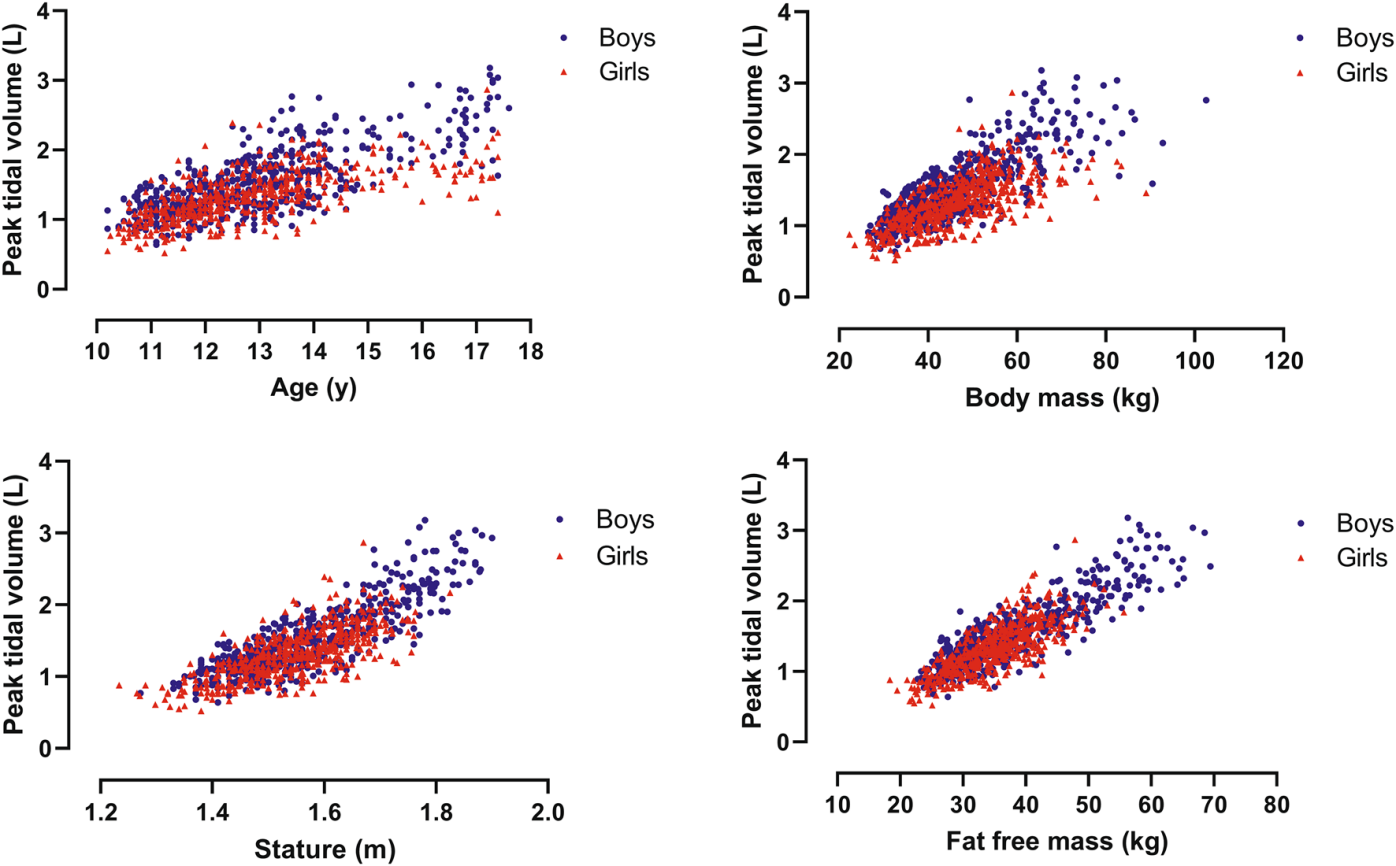
Peak tidal volume in relation to age, body mass, stature, and estimated fat-free mass by sex in 10–17-year-olds

**Fig. 3:**
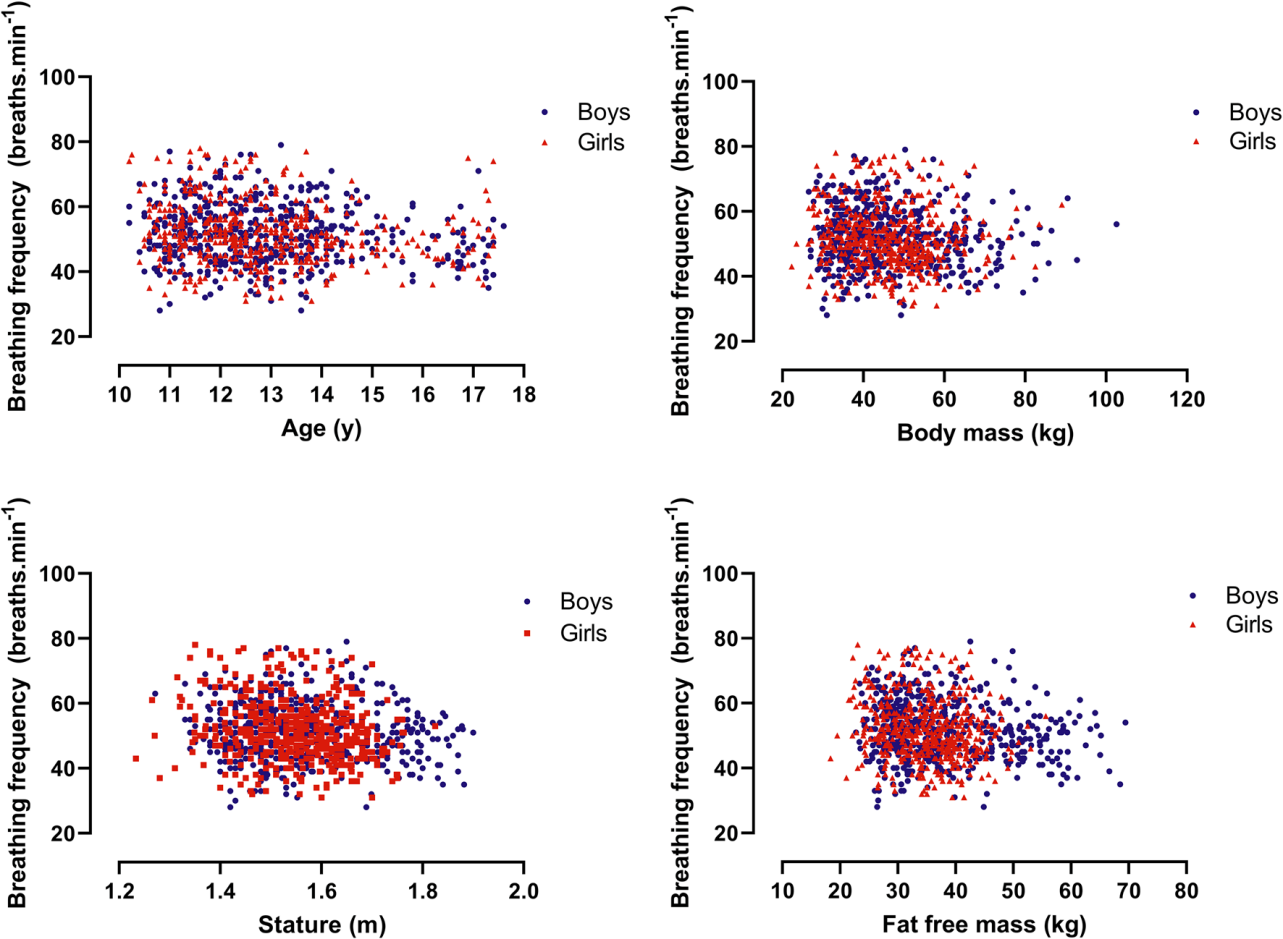
Peak breathing frequency in relation to age, body mass, stature, and estimated fat-free mass by sex in 10–17-year-olds

For descriptive purposes, relationships by sex of peak ventilatory variables with age, body mass, stature, and estimated FFM are illustrated in Figs. 1, 2, 3. As peak *f*_b_ was only weakly associated with age and morphological variables, it was not explored further with multilevel analyses. Tables 1 and 2 present multiplicative allometric models founded on 1053 (550 from boys) determinations of age, body mass, sum of triceps and subscapular skinfolds, stature, and peak ${\dot{\mathrm{V}}}_{\mathrm{E}}$ (Table 1) or peak *V*_T_ (Table 2), respectively.

**Table 1:** Multiplicative allometric models of peak expired ventilation in 10–17-year-olds

	Model 1.1 (boys)		Model 1.2 (boys)		Model 1.3 (girls)		Model 1.4 (girls)	
Response	Log_e_ peak ${\dot{\mathrm{V}}}_{\mathrm{E}}$		Log_e_ peak ${\dot{\mathrm{V}}}_{\mathrm{E}}$		Log_e_ peak ${\dot{\mathrm{V}}}_{\mathrm{E}}$		Log_e_ peak ${\dot{\mathrm{V}}}_{\mathrm{E}}$	
Fixed Part	Estimate	S.E	Estimate	S.E	Estimate	S.E	Estimate	S.E
Constant	1.857	0.163	1.464	0.156	1.966	0.173	1.700	0.181
Age	0.050	0.006	0.025	0.007	0.039	0.007	0.026	0.007
Age^2^	− 0.003	0.001	− 0.002	0.001	− 0.003	0.001	0.004	0.002
Log_e_ Body mass	0.644	0.043	0.876	0.048	0.586	0.045	0.750	0.059
Log_e_ Skinfolds	–		− 0.167	0.021	–		− 0.116	0.028
Log_e_ Stature	–		ns		–		ns	
Random Part								
Level 2								
Variance (cons)	0.010	0.001	0.009	0.001	0.010	0.001	0.009	0.001
Level 1								
Variance (cons)	0.010	0.001	0.010	0.001	0.011	0.001	0.011	0.001
Units Level 2	213		213		207		207	
Units Level 1	550		550		503		503	
− 2*log-likelihood	− 686.496		− 745.887		− 619.641		− 637.150	

Models founded on 1053 (boys, *n* = 550; girls, *n* = 503) determinations of peak expired ventilation

${\dot{\mathrm{V}}}_{\mathrm{E}}$: expired ventilation; skinfolds: sum of triceps and subscapular skinfolds; ns: not significant (*p* > 0.05); –: not entered in model

**Table 2:** Multiplicative allometric models of peak tidal volume in 10–17-year-olds

	Model 2.1 (boys)		Model 2.2 (boys)		Model 2.3 (boys)		Model 2.4 (girls)		Model 2.5 (girls)		Model 2.6 (girls)	
Response	Log_e_ peak *V*_T_		Log_e_ peak *V*_T_		Log_e_ peak *V*_T_		Log_e_ peak *V*_T_		Log_e_ peak *V*_T_		Log_e_ peak *V*_T_	
Fixed part	Estimate	S.E	Estimate	S.E	Estimate	S.E	Estimate	S.E	Estimate	S.E	Estimate	S.E
Constant	− 2.192	0.182	− 2.640	0.169	− 2.350	0.211	− 1.806	0.205	− 2.340	0.204	− 2.171	0.220
Age	0.067	0.007	0.035	0.006	0.029	0.007	0.079	0.007	0.053	0.008	0.049	0.008
Age^2^	− 0.002	0.001	ns		ns		− 0.014	0.002	− 0.008	0.002	− 0.008	0.002
Log_e_ Body mass	0.674	0.048	0.943	0.052	0.745	0.100	0.547	0.053	0.867	0.066	0.728	0.095
Log_e_ Skinfolds	–		− 0.197	0.022	− 0.150	0.030	–		− 0.222	0.030	− 0.182	0.036
Log_e_ Stature	–		–		0.724	0.314	–		–		0.541	0.268
Random Part												
Level 2												
Variance (cons)	0.017	0.002	0.012	0.002	0.012	0.002	0.021	0.002	0.017	0.002	0.017	0.002
Level 1												
Variance (cons)	0.009	0.001	0.008	0.001	0.008	0.001	0.009	0.001	0.008	0.001	0.008	0.001
Units: Level 2	213		213		213		207		207		207	
Units: Level 1	550		550		550		503		503		503	
− 2*log-likelihood	− 680.324		− 752.403		− 757.648		− 580.016		− 630.018		− 634.089	

Models founded on 1053 (boys, *n* = 550; girls, *n* = 503) determinations of peak tidal volume

*V*_T_: tidal volume; skinfolds: sum of triceps and subscapular skinfolds; ns: not significant (*p* > 0.05); –: not entered in model

Table 1 describes multiplicative allometric models of peak ${\dot{\mathrm{V}}}_{\mathrm{E}}$ which illustrate the effects of introducing additional morphological covariates to the baseline model (i.e., Eq. 2) of peak ${\dot{\mathrm{V}}}_{\mathrm{E}}$. Models 1.1 (boys) and 1.3 (girls) show that with age controlled for, there is an additional, significant positive effect of body mass on peak ${\dot{\mathrm{V}}}_{\mathrm{E}}$, in both sexes. Models 1.2 (boys) and 1.4 (girls) show that the introduction of sum of skinfolds as an additional covariate provides models with significantly (*p* < 0.05) better statistical fits for the data than Models 1.1 (boys) and 1.3 (girls), respectively. The introduction of stature into Models 1.2 (boys) and 1.4 (girls) did not produce significantly (*p* > 0.05) better fit models. Age was a positive and significant parameter in all models with the negative age^2^ term, indicating that the size of the age effect decreased as the rate of change in growth reduced. Maturity status (i.e., stages of PH development) had no additional effect on peak ${\dot{\mathrm{V}}}_{\mathrm{E}}$ once age and morphological covariates had been controlled for.

Table 2 is founded on peak *V*_T_ data and the multiplicative allometric models illustrate the effects of introducing additional morphological covariates to the baseline model (i.e., Eq. 2) of peak *V*_T_. Models 2.1 (boys) and 2.4 (girls) show that with age controlled for, there is an additional, significant positive effect of body mass on peak *V*_T_ in both sexes. Models 2.2 (boys) and 2.5 (girls) show that the introduction of sum of skinfolds as an additional covariate provided a significantly (*p* < 0.05) better statistical fit for the data in both sexes. The subsequent introduction of stature in Models 2.3 (boys) and 2.6 (girls), respectively, produced the models with the best statistical fit, in both sexes. Age was a positive and significant parameter in all models with, in Models 2.1, 2.4, 2.5, and 2.6, a negative age^2^ term, indicating that the size of the age effect decreased as the rate of change in growth reduced. Maturity status (i.e., stages of PH development) had no additional significant (*p* > 0.05) effect on peak *V*_T_ once age and morphological covariates had been controlled for.

## Discussion

Directly comparing the magnitude of peak ventilatory variables across studies using different exercise protocols must be done cautiously, but the present data fall well-within the range of values currently presented as CPET paediatric norms (Bongers et al. 2014). In accord with the extant literature (Ruttenfranz et al. 1981; Prioux et al. 1997; Rowland and Cunningham 1997), peak ${\dot{\mathrm{V}}}_{\mathrm{E}}$ and peak *V*_T_ increased in a near-linear manner with each of age, stature, body mass, and FFM with peak *f*_b_ weakly and negatively associated with age and morphological variables, in both sexes.

As with other exercise variables (Welsman and Armstrong 2019; Armstrong and Welsman 2020a), peak ${\dot{\mathrm{V}}}_{\mathrm{E}}$ and peak *V*_T_ are traditionally controlled for changes in growth by simply ratio scaling the data with body mass and reporting peak ${\dot{\mathrm{V}}}_{\mathrm{E}}$ in L·kg^−1^·min^−1^ and peak *V*_T_ in L·kg^−1^, respectively. The fallacy of ratio scaling either peak ${\dot{\mathrm{V}}}_{\mathrm{E}}$ or peak *V*_T_ with body mass to control for growth is clearly demonstrated in the baseline models [Models 1.1 (boys), 1.3 (girls), 2.1 (boys), and 2.4 (girls)], where the body mass exponents for peak ${\dot{\mathrm{V}}}_{\mathrm{E}}$ and peak *V*_T_, respectively, are 0.64 and 0.67 (boys) and 0.59 and 0.55 (girls). In all cases, an exponent of 1.0 which is a statistical requirement in ratio scaling falls outside the 95% confidence limits. In contrast to previous studies using ratio scaling of body mass (e.g., Åstrand 1952; Rowland and Cunningham 1997; Giardini 2011), the baseline models show that with body mass appropriately controlled for, both peak ${\dot{\mathrm{V}}}_{\mathrm{E}}$ and peak *V*_T_ increase with age, in both sexes.

Multiplicative allometric modelling allows individual growth trajectories to be modelled and the presentation of the incremental models in Tables 1 and 2 is designed to evidence the significance of concurrent changes in several covariates in explaining the development of peak ventilatory variables. Table 1 presents sex-specific models of peak ${\dot{\mathrm{V}}}_{\mathrm{E}}$, with Models 1.2 (boys) and 1.4 (girls) being the best fit models for age and morphological covariates. The entry of stature into either Model 1.2 (boys) or Model 1.4 (girls) did not provide a significantly better fit model for peak ${\dot{\mathrm{V}}}_{\mathrm{E}}$ in either sex. This is in agreement with previous reports, where multiplicative allometric modelling showed FFM (i.e., body mass and sum of skinfolds) to be the principal morphological influence on the development of a range of other peak exercise variables, such as peak $\dot{\mathrm{V}}{\mathrm{O}}_{2}$ (Armstrong and Welsman 2019b), peak power output (Armstrong et al. 2019), peak cardiac output (Armstrong and Welsman 2020b), and peak stroke volume (Armstrong and Welsman 2019c). As peak ${\dot{\mathrm{V}}}_{\mathrm{E}}$ only reaches ~ 70% of maximal voluntary ventilation in youth (Nixon 2018), increases in peak ${\dot{\mathrm{V}}}_{\mathrm{E}}$ during growth and maturation are primarily driven by the concurrent development of increases in carbon dioxide output, muscle lactate, and peak $\dot{\mathrm{V}}{\mathrm{O}}_{2}$. Peak ${\dot{\mathrm{V}}}_{\mathrm{E}}$ is therefore likely to be more closely related to FFM as a surrogate of metabolically active muscles than to parameters of body size (Rutenfranz et al. 1981; Mercier et al. 1991; Prioux et al. 1997).

In both sexes, with FFM controlled for, age remained a significant covariate of peak ${\dot{\mathrm{V}}}_{\mathrm{E}}$ but, in accord with previous studies of cardiopulmonary variables (Armstrong and Welsman 2019b, 2019c,2020b), maturity status did not make an additional contribution to explaining the development of peak ${\dot{\mathrm{V}}}_{\mathrm{E}}$ in either sex. The explanation for a non-significant, additional contribution of maturity status to explaining peak ${\dot{\mathrm{V}}}_{\mathrm{E}}$ is likely to lie in the sex-specific, maturation-driven development of FFM masking any independent effects. The development of FFM is governed by age and the timing and tempo of maturation which is evidenced by boys experiencing a spurt resulting in an ~ 83% increase in FFM over a 4-year period centred on peak height velocity (PHV) and girls experiencing a smaller and shorter spurt resulting in a ~ 31% increase in FFM over a 2-year period centred on PHV (Baxter-Jones et al. 2003).

Models 2.1 (boys), 2.2 (boys), 2.4 (girls), and 2.5 (girls) in Table 2 show the development of peak *V*_T_ to initially show a similar pattern to the development of peak ${\dot{\mathrm{V}}}_{\mathrm{E}}$, in both sexes. However, in contrast to ${\dot{\mathrm{V}}}_{\mathrm{E}}$, the introduction in models 2.3 (boys) and 2.6 (girls) of stature in addition to age, body mass, and skinfold thicknesses provided the statistical best fit models of peak *V*_T_, in both sexes. Maturity status did not make a significant, additional contribution to explaining the development of peak *V*_T_.

There are no previous studies of the development of peak *V*_T_ in relation to concurrent changes in age, body mass, skinfold thicknesses, and stature to directly compare with the current study. However, it has been consistently reported that lung volumes develop in accord with greater lung dimensions, which in turn are related to increases in stature (Rutenfranz et al. 1981; Mercier et al. 1991; Prioux et al. 1997). Mercier et al. (1991) reported estimated FFM to be more strongly related to peak *V*_T_ than stature but, in contrast to the present study, when either stature or estimated FFM was controlled for using ‘simple allometry’, peak *V*_T_ was reported to not change significantly with age. The present study is the first to demonstrate that concurrent changes in stature, body mass, and skinfold thicknesses are all significant, independent explanatory covariates of the development of peak *V*_T_ and that with stature, body mass, and skinfold thicknesses controlled for, peak *V*_T_ still increases with age.

Collectively, the models in Tables 1 and 2 demonstrate the limitations of interpreting developmental changes in peak ventilatory variables with reference solely to chronological age or a single morphological covariate as practised in current CPET norm tables (e.g., Bongers et al. 2014). The multiplicative allometric models expose the fallacy of ratio scaling peak ventilatory variables with body size and evidence the contribution of sex-specific, concurrent changes in both age and morphological covariates in explaining the development of peak ${\dot{\mathrm{V}}}_{\mathrm{E}}$ and peak *V*_T_. The multiplicative allometric equations presented herein can be used to estimate the peak ${\dot{\mathrm{V}}}_{\mathrm{E}}$ and peak *V*_T_ of healthy 10–17-year-olds for comparative purposes, but population-specific studies are required to better inform the interpretation of data from national and international paediatric pulmonary function laboratories.

## Strengths and limitations

FFM includes tissues not involved in exercise and a limitation of the present study is the use of body mass and sum of skinfolds as a surrogate for FFM and indirectly for active muscle mass during exercise. Ideally, active muscle mass would be directly determined on each test occasion, but this is not currently feasible in pediatric exercise studies. Moreover, ‘direct’ measures of the FFM of children and adolescents can also be criticised as it has recently been demonstrated that they vary widely across laboratory techniques (Ferri-Morales et al. 2018). Maturity status was assessed using stages of PH development (Tanner 1962) and, as would be expected with 10–17-year-olds, all stages of PH development were present in the data. It is acknowledged, however, that different biological systems mature at different rates and no single method of assessment presents a complete description of the process, although correlations between indicators of maturity status are generally moderate-to-high (Malina et al. 2004).

The principal strength of the present study lies in the adoption of multiplicative allometric modelling to analyze 1053 determinations of the peak ${\dot{\mathrm{V}}}_{\mathrm{E}}$ and peak *V*_T_ of 10–17-year-olds. All participants did not have full data sets, but multiplicative allometric modelling accommodates variation in both the number of observations per individual and the temporal spacing of the observations. The effects of age, body mass, skinfold thicknesses, and stature, with reference to maturity status, are partitioned concurrently within an allometric framework to provide unique insights into the contribution of changes in age and morphological covariates to the development of peak ventilatory variables.

## Conclusions

The descriptive data from 1053 determinations of peak ${\dot{\mathrm{V}}}_{\mathrm{E}}$, peak *V*_T_, and peak *f*_b_ show that (i) peak ${\dot{\mathrm{V}}}_{\mathrm{E}}$ and peak *V*_T_ increase in a near-linear manner with each of age, body mass, stature, and estimated FFM; and, (ii) peak *f*_b_ remains relatively stable with a slight but consistent decline with age and body size, so that increases in peak ${\dot{\mathrm{V}}}_{\mathrm{E}}$ are solely due to increases in peak *V*_T_.

The multiplicative allometric models evidence that (i) body mass and sum of skinfolds (as a surrogate for FFM) are the most influential morphological covariates in explaining the development of peak ${\dot{\mathrm{V}}}_{\mathrm{E}}$ in both sexes; (ii) concurrent changes in age, body mass, sum of skinfolds, and stature are all significant, explanatory covariates of the development of *V*_T_, in both sexes; and, (iii) CPET paediatric norms for peak ventilatory variables based solely on age or ratio-scaled body mass or stature are not tenable.

## Abbreviations

CPET: Cardiopulmonary exercise test
FFM: Fat-free mass
PHV: Peak height velocity
$${\dot{\mathrm{V}}}_{\mathrm{E}}$$: Expired ventilation
*V*_T_: Tidal volume
*f*_b_: Breathing frequency
PH: Pubic hair
S.E.: Standard error
$$\dot{\mathrm{V}}{\mathrm{O}}_{2}$$: Oxygen uptake
